# Introduction to the Special Issue on “State-of-the-Art Sensor Technology in Japan”

**DOI:** 10.3390/s100504756

**Published:** 2010-05-10

**Authors:** Yoshiteru Ishida

**Affiliations:** Department of Knowledge-Based Information Engineering, Toyohashi University of Technology; 1-1, Tempaku, Toyohashi, Aichi 441-8580, Japan; E-Mail: ishida@tutkie.tut.ac.jp; Tel.: +81-532-44-6895; Fax: +81-532-44-6873

The combination of sensing technology with information and communication technology (ICT) could serve both as global eyes that monitor the environment for environmental issues, and as local eyes that monitor humans for aging society issues. System technology is also required to form such global and local eyes. This special issue, “State-of-the-Art Sensor Technology in Japan”, contains articles and reviews related to the monitoring of humans and the environment, and the integration of sensor systems.

## Sensing Technologies: A Perspective for Japan

It is often pointed out that Japan faces both human issues and environmental issues, the former originating from the domestic situation and the latter from international circumstances. In terms of human issues, Japan’s demographics are rapidly changing as the population ages. Japan has become a nation of elderly people, with the proportion of people over sixty-five exceeding 21% in 2007. As for environmental issues, Japan needs to play a leadership role, not only in international politics, but also in industrial and academic fields.

Sensing technology and information and communication technology (ICT) can help tackle these two issues. For example, sensing technology could play a primary role in gathering data into the global network. Environmental issues together with ICT [1,2] and aging society issues [2] have often been pointed out, and we focus on them from the viewpoint of sensing technologies. Although security is also important [2,3], we must leave it to a future special issue.

Because sensing technology is related to the gateway to the network, we also examine intelligent information processing to realize sensor fusion, intelligent sensing, sensor networks and sensor systems. Both environmental monitoring and human monitoring require flexibility and adaptation, hence a third category is the integration of sensor systems and networks that involve machine learning and adaptation technology.

## Sensing Technology for Human Monitoring

Sensors for monitoring humans and bio-systems require several technologies. Optic technologies [4,5] and carbon nanotubes [6] are promising and essential technologies, and have been attracting much attention not only for biosensors, but also other areas including damage detection [7]. Other technologies such as mass spectrometry [8] have been attracting attention for biosensors. CMOS technology [9,10] allows small size and low power consumption, enabling the sensors to be implanted in humans and animals.

For the aged society, human monitoring in pioneering fields is required, such as oropharyngeal swallowing [11] and finger tapping movement [12], as well as the monitoring of vital signs [13] and specific activities such as farming operations [14]. Wearable sensors are also needed for monitoring elderly people.

Sensing technology can also be used to understand human sensors [15] or even to support human sensors [9]. Finally, further studies on sensing technology in relatively unexplored areas such as the senses of smell [15,16] and taste [17] are needed.

## Sensing Technology for Monitoring the Environment

Sensing technology for monitoring the environment requires global monitoring involving sensor systems (including sensor networks), as described in the next section, and infrastructure such as satellites [18–20] and submarine stations [21].

The specific sensing technologies required depend on the target, which includes land surface temperature [18], near-surface seawater temperature [22], seismic and tectonic activities [21], and aeolian sand transport [23]. Even when monitoring the same or similar measurements, regional characteristics mean that there are region-specific problems in monitoring, so field studies will be essential [19,21].

Global-scale monitoring is important for environmental monitoring, but it involves systems science and technologies other than earth science and sensing technologies. The difficulty of environmental monitoring comes from not only its large scale but also its complex system aspects (entanglement of cause and effect; mixture of slow and rapid processes; inextricable linkage between humans and natural environment).

## Intelligent Information Processing for Sensor Systems

Sensor system technologies need to involve many technologies to allow the integration of multiple and multimodal sensors (*i.e.*, sensor fusion), a network of autonomous sensor units (*i.e.*, sensor network), and post-processing of sensed data (*i.e.*, intelligent sensing). The next generation of sensor system technologies must be more flexible to attain more efficient, intelligent and autonomous sensor systems.

Fortunately, intelligent information processing technologies such as artificial intelligence (AI) and machine learning (ML) are sufficiently mature to provide such flexibility. These include feature extraction [24]; classification of sensed events [12,19,25]; cooperative communications in networking sensor nodes [26]; adaptability to deal with the trade-off between false alarms and missed alarms [27]; and adaptability to allow automatic weighting of important and credible sensors [28].

## Sensing Technology: A Challenge

Globalization has been driven by the explosive expansion of the information network involving the Internet, satellites, and sensor networks. Since the network now covers the globe, the next stage is to organize the functions and integration for each level, place, and situation.

Network expansion and globalization can be considered to be the development phase of the central nervous system of the earth. In the next phase, the network should be integrated with sensor and motor systems.

Sensing technology R&D requires many disciplines: sciences and humanities; science and engineering; and many sections within each branch of science and engineering. Although it is usually convenient and necessary to work within a discipline, it is also necessary to conduct studies spanning different fields. Sensing technology and science inherently require cross-sectional studies, and articles of this special issue indeed show the importance of such studies.

Although globalization with ICT encourages such cross-sectional studies by enabling a cooperative R&D involving intensive computing and networking (as found within the frameworks of *e-Science* and *cyberinfrastructure*), it is a challenge to manage and implement them for the cross-sectional studies of sensing technology.
